# Rethinking antisense oligonucleotide therapeutics for amyotrophic lateral sclerosis

**DOI:** 10.1002/acn3.52234

**Published:** 2024-10-29

**Authors:** Daisuke Ito, Kensuke Okada

**Affiliations:** ^1^ Memory Center Keio University School of Medicine Tokyo Japan; ^2^ Department of Neurology Keio University School of Medicine Tokyo Japan

## Abstract

Antisense oligonucleotides, which are used to silence target genes, are gaining attention as a novel drug discovery modality for proteinopathies. However, while clinical trials for neurodegenerative diseases like amyotrophic lateral sclerosis have been conducted in recent years, the results have not always been favorable. The results from a Phase III trial of the antisense oligonucleotide, that is, tofersen, which targets *SOD1* mRNA, showed decreased levels of cerebrospinal fluid SOD1 and plasma neurofilament light chain but no improvements in primary clinical endpoint. Moreover, case reports pertaining to patients with amyotrophic lateral sclerosis carrying *FUS* and *C9orf72* mutations who received antisense oligonucleotide‐based treatments have demonstrated a notable reduction in the targeted protein (thus providing the proof of mechanism) but with no discernible clinical benefits. There are several possible reasons why antisense oligonucleotides knockdown fails to achieve proof of concept, which need to be addressed: on‐target adverse effects resulting from the loss of function of target gene and irreversible neuronal death cascade due to toxic protein accumulation, among other factors. This review provides an overview of the current status and discusses the prospects of antisense oligonucleotides treatment for amyotrophic lateral sclerosis.

## Introduction

Amyotrophic lateral sclerosis (ALS) is a fatal neurological disease characterized by progressive degeneration of upper and lower motor neurons. An estimated 10% of ALS cases are due to single gene mutations with a Mendelian inheritance pattern.^1^ A major discovery in 2006 was the identification of abnormal accumulation of the RNA binding protein, TAR DNA binding protein 43 (TDP‐43), and frontotemporal dementia (FTD), as a pathological hallmark of ~90% of sporadic ALS.^2^, ^3^ Seven ALS‐related molecules are RNA‐binding proteins in which mutations are known to cause ALS. These include TDP‐43 itself; FUS; TATA box‐binding protein‐associated factor 15 (TAF15); Ewing sarcoma breakpoint region 1 (EWSR1), and the heterogeneous nuclear ribonucleoproteins (hnRNPs) hnRNPA1, hnRNPA2B1, and T‐cell intracytoplasmic antigen (TIA1).^4^ Notably, the most common ALS mutation, the large hexanucleotide (GGGGCC) repeat expansion in the chromosome 9 open reading frame 72 (C9orf72) gene, also leads to TDP‐43 accumulation in motor neurons. Thus, major ALS can be considered an RNA‐binding proteinopathy, and it has been suggested that RNA dysregulation is key to its pathomechanism. In contrast, no aberrant RNA‐binding proteins have been observed in ALS with the first ALS‐causing gene identified, superoxide dismutase 1 (SOD1) mutations, suggesting that the molecular pathogenesis is basically different from that of major ALS with RNA‐binding proteinopathy.

Only riluzole has been approved by the US Food and Drug Administration (FDA), the European Medicines Agency, and other countries for the treatment of ALS with RNA‐binding proteinopathy. However, this drug has a limited effect, only extending life expectancy by 2 months. Edaravone is approved in several countries, except the EU, but long‐term observations in Germany recently showed no clinical benefit.^5^ The development of new drugs is therefore urgently needed.

Nucleic acid therapeutics (NATs), which target RNA rather than proteins, are emerging as the next generation of treatments. They include antisense oligonucleotides (ASOs), small interfering RNAs (siRNAs), microRNAs (miRNAs), aptamers, and ribozymes that are involved in controlling disease‐related protein expression through mRNA degradation or translational modulation. Synthetic single‐stranded deoxyribonucleotides, known as ASOs, inhibit translation by disrupting ribosome assembly, promoting mRNA degradation or inducing splice‐switching. Their small molecular weight and ability to reach the nucleus enable them to knockdown a broad range of molecules, including mRNAs, pre‐miRNAs, and miRNAs.

The clinical trials to assess the efficacy of patisiran, a small interfering RNA, and inotersen, an antisense oligonucleotide (ASO) targeting transthyretin (*TTR*) mRNA in hereditary ATTR amyloidosis, showed positive results, as expected.^6^, ^7^ Hopes are high for managing central nervous system (CNS) proteinopathies through knockdown approaches targeting specific genes, and ASOs are viewed as promising disease‐modifying agents.^4^, ^8^ Despite numerous relevant clinical trials conducted against neurodegenerative diseases in recent years, the outcomes have not always been favorable (Tables 1 and 2).

**Table 1:** ASO therapies and clinical trials in ALS.

Gene	Drug	Study phase	Primary/secondary outcome	Study completion/outcome	Identifier/Status	Phenotypes in gene deficiency
RNase H‐mediated degradation						
*SOD1*	Tofersen (BIIB067)	FDA‐accelerated approval	Change from baseline in ALSFRS‐R total score at Week 28	No improvement in clinical end points but reduced plasma NLF	NCT02623699 /completed	Progressive distal motor axonopathy in an age‐dependent manner in mice. Progressive motor deficits, spastic quadriplegia, and axial hypotonia in human child
		Phase III	Emergence of clinically manifested ALS within 12 months	2027/8/7	NCT04856982/recruiting	
*C9orf72*	BIIB078	Phase I	Incidence of AEs and SAEs/ALSFRS‐R total score	No clinical benefit (ALSFRS‐R)	NCT03626012 /discontinued	Altered immune responses in macrophages and microglia and exacerbated neurodegeneration in C9‐model mice
	Afinersen	Case report	ALSFRS‐R total score and CSF polydipeptides (polyGP) concentration	Reduced CSF polyGP but no change of ALSFRS‐R	IND application (IND141673)	
	WVE‐004	Phase I/II	Incidence of AEs/CSF polyGP concentration	Reduction in CSF polyGP but no clinical benefit (ALSFRS‐R or CDR)	NCT04931862/discontinued	
*FUS*	Ulefnersen (ION363, Jacifusen)	Phase I–III	ALSFRS‐R total score and ventilation assistance‐free survival	2028/3/1One case with P525L mutation: No improvement natural history of ALS with P525L. But FUS‐pathological hallmarks were reduced at autopsy.	NCT04768972/recruiting	Constitutive knockout is perinatal lethal but has no effect on motor neuron survival in mice
*ATXN2*	BIIB105	Phase I/II	Incidence of AEs	Reduction in CSF ATXN2 but no reduction in NfL or clinical benefit	NCT04494256/recruiting	Obesity, no neurological problems in mice
*CHCHD10*	BIIB105	Single case	ALSFRS‐R total score and ALS Cognitive Behavioral Screen	2025/4/1	NCT06392126/recruiting	Knockout mice have no gross phenotypes, no mitochondrial abnormalities
Blocking a cryptic‐exon splice						
*STMN2*	QRL‐201	Phase I	Incidence of AEs	2025/5/6	NCT04494256/discontinued	Heterozygous *STMN2* knockout mice show motor neuropathy and NMJ denervation

AEs, adverse events; ALS, amyotrophic lateral sclerosis; ALSFRS‐R, Amyotrophic Lateral Sclerosis Functional Rating Scale‐Revised Scores; CDR, clinical dementia rating; CSF, cerebrospinal fluid; NfL, neurofilament light chain, NMJ, neuromuscular junction.

**Table 2:** ASO therapies and clinical trials in other major neurodegenerations.

Disease/gene	Drug	Study phase	Primary/secondary outcome	Study completion/outcome	Identifier/status	Phenotypes in gene deficiency
RNase H‐mediated degradation						
Huntington's disease/*HTT*	WVE‐120101	Phase I/II	TFC in UHDRS	No change in CSF and Htt	NCT03225833/discontinued	Progressive motor deficits and an anxious phenotype in mice
	WVE‐120102	Phase I/II		No change in CSF and Htt	NCT03225846	
	WVE‐3	Phase I/II	Incidence of AEs	2023/6/1	NCT05032196/recruiting	
	Tominersen	Phase III	Composite UHDRS	Worsening clinical severity on high dosing	NCT03761849	
		Phase II	Incidence of AEs/cUHDRS	2027/1/4	NCT05686551/recruiting	
Parkinson's disease/*α‐Synuclein*	ION464 (BIIB101)	Phase I	Incidence of AEs/alpha‐synuclein in CSF	2025/12/1	NCT04165486/recruiting	Synaptic vesicle depletion and an anxious phenotype in mice
Parkinson disease/*LRRK2*	BIIB094	Phase I	Incidence of AEs	2023/12/2	NCT03976349/recruiting	Kidney degeneration and abnormal exploratory activity in mice
Alzheimer's disease /*MAPT*	IONIS MAPTRx	Phase I/II	Incidence of AEs	Dose‐dependent reduction in CSF total‐tau	NCT03186989/completed	Short‐term memory deficits and increased locomotor activity in mice
	NIO75	Phase I	Incidence of AEs	2024/11/12	NCT04539041/recruiting	
		Phase I/II	Change in cerebrospinal total tau/incidence of AEs	2024/10/30	NCT05469360/recruiting	

AEs, adverse events; CSF, cerebrospinal fluid; HD, Huntington disease; Htt, Huntingtin; LRRK2, leucine rich‐repeat kinase 2; TFC, total functional capacity, UHDRS, Unified Huntington's Disease Rating Scale.

Leading clinical trials to examine the potential of three huntingtin (HTT)‐lowering ASOs in Huntington disease were recently halted. In early 2021, Wave Life Sciences discontinued two single‐nucleotide polymorphism‐selective ASO studies, WVE‐120101 and WVE‐120102, that failed to consistently lower mutant HTT protein in the cerebrospinal fluid.^9^ In a Phase III clinical trial with tominersen, despite a dose‐dependent reduction of mutant HTT in the cerebrospinal fluid, a clear clinical benefit was not recorded, and clinical scores worsened in participants who received high drug doses.^10^ Whether the worsening clinical severity was caused by off‐target effects or neuroinflammation due to the ASO intrathecal injection or by loss of on‐target HTT activity is not yet known. In rodents, reduction in HTT expression in conditional knockout mice resulted in progressive motor deficits and an anxious phenotype in adulthood,^11^, ^12^ suggesting that on‐target toxicity caused by the loss of wild‐type HTT function with ASO treatment is critical in clinical trials.

**Figure 1:**
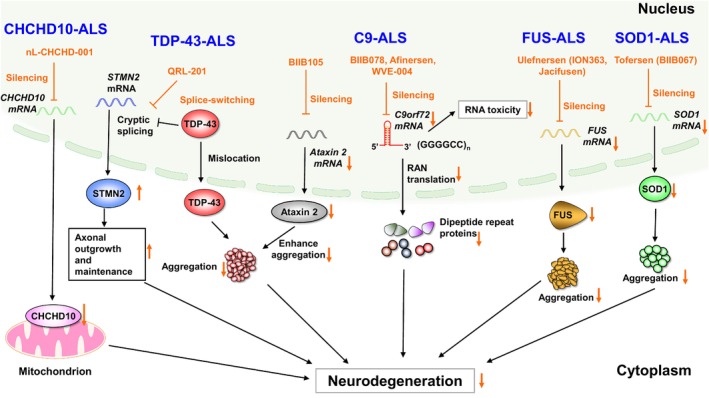
The promising mechanisms of ongoing ASOs in the pathophysiology of ALS. Silencing of target genes by tofersen, ulefnersen, BIIB078, and nL‐CHCHD‐001 reduces toxic protein aggregation of SOD1, FUS, dipeptide repeat proteins, and CHCHD10, respectively. In TDP‐43‐ALS, silencing of the modulator of TDP‐43, ataxin2, by BIIB105 reduces TDP‐43 aggregation. A splicing switch of *STMN2* using QRL‐201 results in the production of a full‐length STMN2 and normal axonal outgrowth. Abbreviations: ALS, amyotrophic lateral sclerosis; ASO, antisense oligonucleotides; C9‐ALS, ALS with C9orf72 mutation; FUS‐ALS, ALS with FUS mutation; SOD1‐ALS, ALS with SOD1 mutation; CHCHD10‐ALS, ALS with CHCHD10 mutation.

Meanwhile, in a preclinical study of ALS, suppression of toxic TDP‐43 expression in doxycycline‐regulatable mice after onset of motor dysfunction resulted in rapid rescue of the motor phenotype and lifespan extension, indicating that removal of toxic TDP‐43 can aid functional recovery.^13^ Therefore, ASO‐mediated knockdown of target genes may be a relevant and promising treatment for ALS (Fig. 1). This review summarizes the results of recent ALS–ASO clinical trials and discusses the prospects and issues that need to be addressed.

## ALS with *SOD1* Mutation

The results of a Phase III trial of the ASO tofersen, which targets *SOD1* mRNA, were disclosed in 2022.^14^ In this study, a total of 108 participants were enrolled, with 60 categorized into the faster progression subgroup based on both the *SOD1* mutation type and the estimated slope of Revised ALS Functional Rating Scale (ALSFRS‐R). The primary efficacy endpoint assessed was the change from baseline in ALSFRS‐R at 28 weeks in the faster progression subgroup. Tofersen reduced SOD1 protein levels in the cerebrospinal fluid (geometric mean ratio: tofersen vs. placebo: 0.62) in all participants, indicating proof‐of‐mechanism. The levels of plasma neurofilament light chain (NfL), a marker of axonal injury, decreased by 60% in the treated group and increased by 20% in the placebo group. However, no significant differences in the primary efficacy endpoint in the faster‐progression subgroup were recorded (between‐group difference, 1.2; *p* = 0.97), although a desirable trend was observed for secondary and exploratory endpoints (motor and respiratory function and quality of life). Most adverse events were mild to moderate. Around 7% of participants receiving tofersen experienced serious neurological adverse events, including myelitis, chemical or aseptic meningitis, and lumbar radiculopathy. Post hoc analyses of open‐label extension (OLE) at 52 weeks revealed clinical benefits in several parameters between early‐start tofersen (*n* = 72) and delayed‐start tofersen (*n* = 36). These included improvements in the ALSFRS‐R total score (adjusted mean difference 3.5 [95% CI, 0.4–6.7]; *p* = 0.0272), forced vital capacity (9.2 [95% CI, 1.7–16.6]; *p* = 0.0159), handheld dynamometry (0.28 [95% CI, 0.05–0.52]; *p* = 0.0186). The FDA granted accelerated approval to tofersen based on its ability to reduce the levels of the biomarker NfL and the OLE data. Recent real‐world evidence in Germany confirms both a reduction of NfL serum levels and favorable safety and tolerability.^15^ Thus, while challenges remain because it takes a few years to confirm the clinical benefit from changes in a fluid biomarker, therapeutic research on tofersen is making steady progress.

The next focus for tofersen therapy is to evaluate the effects in presymptomatic carriers of SOD1 mutation, and the ATLAS study (NCT04856982) has been designed to investigate whether tofersen can delay the emergence of clinically manifested ALS.^14^ Participants in this study must carry a rapid progressive *SOD1* variant (mostly p.Ala5Val), be clinically presymptomatic for ALS, and have a plasma NfL level below the predefined threshold of 44 pg/mL, which minimizes the false‐positive rate. During a natural history run‐in period, if a participant is found to have an elevation in plasma NfL level exceeding the predefined threshold (plasma NfL level ≥ 44 pg/mL and an increase of ≥10 pg/mL from baseline) while remaining presymptomatic, they become eligible to participate in a randomized, placebo‐controlled period. The primary endpoint is emergence of clinically manifested ALS within 12 months.

In a rodent model, homozygote SOD1 knockout mice were initially reported to lack the pathological phenotype, but progressive distal motor axonopathy developed in an age‐dependent manner.^16^

Notably, in several children, homozygous loss‐of‐function variants of *SOD1* have been observed to cause progressive motor deficits, spastic quadriplegia, and axial hypotonia,^17^, ^18^, ^19^ suggesting that SOD1 contributes to neuronal homeostasis and maintenance. There was an up to 50% decrease in SOD1 protein expression and enzyme activity in the heterozygous family members, who were asymptomatic, indicating that a 40% reduction in the cerebrospinal fluid by tofersen may be tolerated.

Treatment with edaravone, a radical scavenger approved by the FDA, indicates that the presence of radical scavengers is more important in patients with ALS than in non‐affected people. Owing to the size and complexity of the human brain, ASO distribution by intrathecal injection is uneven, and enzyme activity may be less than 50%, depending on the CNS region and cell type. Given the phenotype of SOD1 deficiency in human and mice, long‐term reduction of SOD1 levels requires careful evaluation.

## ALS with *FUS* Mutation

Ulefnersen (also known as ION363 or jacifusen) is an ASO designed to suppress the expression of *FUS*, the third most common gene in familial ALS. A 25‐year‐old patient with the FUS–P525L mutation^20^ presented with respiratory failure requiring noninvasive positive‐pressure ventilation. Her twin sister, also with the same mutation, was diagnosed at 11 years old and survived for 6 years with a ventilator before this drug was developed. After starting ulefnersen treatment (total of 12 infusions over 10 months), the ALSFRS‐R score seemed to slowly decrease; however, respiratory failure and dysphagia progressed, and the patient died from complications approximately 1 year after initiating treatment and 18 months after disease onset. Details on complications are not currently available. Notably, immunohistochemical staining and immunoblotting showed that the abnormal accumulation of the FUS protein in the spinal cord was markedly reduced, indicating proof‐of‐mechanism. However, there was no improvement compared to the natural history of ALS with this mutation, which an average of 13.8 months from the onset to respiratory failure.^21^ This pathological–clinical finding resembles a previous case report of ALS with the SOD mutation treated with gene therapy using adeno‐associated virus containing an anti‐SOD1 microRNA, which also showed a dramatic reduction of SOD1 levels in the spinal cord, but the patient died from respiratory arrest without showing any functional improvement.^22^

A Phase III clinical trial of ulefnersen is currently underway, involving up to 95 participants. In this study, participants will be randomized in a 2:1 ratio to receive a multiple‐dose regimen of ulefnersen for 60 weeks, followed by an open‐label extension for 84 weeks. The primary outcome is the change from baseline in functional impairment, assessed through a joint rank analysis of the combined assessment of ALSFRS‐R, time to rescue or discontinuation due to functional deterioration, and ventilator‐assisted survival. This study is expected to be completed in March 2024.

In a rodent model, constitutive knockout of FUS (FUS−/−) resulted in perinatal lethality despite normal motor neuron development.^23^ Conditional FUS knockout mutants revealed that postnatal motor neuron‐selective elimination of FUS has no effect on motor neuron even at 1 year of age.^24^ FUS is pivotal for DNA repair, RNA splicing, RNA translation, and RNA transport,^25^ and the effects of long‐term reduction of FUS levels, including in glial cells, remain unknown.

## C9orf72 in ALS/Frontotemporal Dementia (C9‐ALS/FTD)

To date, three clinical trials using ASO therapies targeting C9orf72 have been conducted (WVE‐004, BIIB078 [tadnersen], and afinersen). However, BIIB078 and WVE‐004, in Phase I/II, showed no clinical benefit, and their development was discontinued.^26^, ^27^

Although the high dose of BIIB078 did not result in clinical benefits (as assessed by ALSFRS‐R, Slow Vital Capacity, Hand‐Held Dynamometry, and the Iowa Oral Pressure Instrument), it showed a trend toward a greater decline compared to the placebo.^26^ In WVE‐004 for C9‐ALS/FTD, sustained reductions in poly(GP), a pharmacodynamic biomarker indicative of toxic polydipeptides translated from the expanded GGGGCC repeat, were observed from baseline, with a maximal mean reduction of approximately 50%. However, despite this, no clinical benefit (as assessed by ALSFRS‐R and Clinical Dementia Rating Dementia Staging Instrument plus National Alzheimer Coordination Center) was observed at 24 weeks, and reductions in poly(GP) were not found to be associated with functional outcomes.^27^

For afinersen, first‐in‐human data have been published.^28^ A 60‐year‐old patient with a *C9ORF72* GGGGCC expansion of approximately 2400 repeats was treated via intrathecal administration for 14 months (a total of eight administrations). Although the levels of polyGP in the cerebrospinal fluid decreased to 20%, NfL levels increased fivefold inversely. Additionally, the patient's ALSFRS‐R remained unchanged for 10 months.

Although C9orf72 knockout in mice originally led to altered immune responses in macrophages and microglia without overt neuronal deficits,^29^ C9orf72‐depleted microglia in aged mice enhanced cortical synaptic pruning.^30^ C9orf72 loss or haploinsufficiency in a C9‐ALS/FTD mouse model (transgenic mice that carry a mutant C9ORF72 bacterial artificial chromosome) exacerbates motor behavior deficits.^31^ This observation indicates that altered microglial function due to decreased C9orf72 expression contributes to neurodegeneration.

## Moderator of TDP‐43, Ataxin 2 (ATXN2)

Because several lines of evidence suggest that loss of nuclear TDP‐43 function contributes to disease pathogenesis in TDP‐43 proteinopathy,^32^, ^33^, ^34^ ASO knockdown against TDP‐43 is not simply indicated. Thus, moderators of TDP‐43 could be a more appropriate molecular target for ASO therapy in ALS.

Individuals with spinocerebellar ataxia 2 typically exhibit a relatively high CAG repeat expansion (over 34) in the *ATXN2* gene, whereas healthy individuals typically have fewer than 26 repeats. The risk for ALS increases with intermediate‐length CAG expansions (30–33 glutamines) in *ATXN2*, according to the study by Elden et al.^35^ Using an ASO–ATXN2, this group found that motor function and lifespan were improved in transgenic TDP‐43 mice.^36^

The ATXN2‐knockout mice exhibit no significant neurological problems, except obesity with insulin resistance and dyslipidemia,^37^, ^38^ suggesting that ATXN2 plays a minor role in the nervous system. These results led to the initiation of a Phase I clinical trial (NCT04494256) to evaluate the effects of ASO administration targeting ATXN2 (BIIB10). Portions of this trial are focused on individuals with medium‐length CAG expansions in ATXN2. Unfortunately, in May 2024, Biogen and Ionis announced that BIIB10 demonstrated a significant reduction in ATXN2 protein in the CSF but no reduction in NfL or clinical benefit over the 6‐month placebo‐controlled period, resulting in the discontinuation of development.

## TDP‐43‐Regulated *Stathmin 2* ( *STMN2* ) and *UNC13A* mRNA

To gain insights, a comprehensive search for TDP‐43‐regulated mRNAs identified two critical genes: *STMN2*, which mediates neurite extension and maintains neuromuscular junctions,^32^, ^33^ and *UNC13A*, which mediates calcium‐triggered synaptic vesicle release.^34^

*STMN2*‐knockout heterozygous mice display a slowly progressive, motor‐selective neuropathy and denervation at the neuromuscular junction.^39^ Mice with UNC13A deficiency experience perinatal death and display total arrest of synaptic transmission due to a complete loss of fusion‐competent synaptic vesicles.^40^ Homozygous loss‐of‐function mutation of *UNC13A* in human causes microcephaly, cortical hyperexcitability, and fatal myasthenia.^41^

Nuclear TDP‐43 loss in patients with ALS results in incorporation of a premature polyA tail in *STMN2* and inclusion of a cryptic exon in the *UNC13A* transcript, leading to reduced function in these proteins. Clinical trials are planned to correct aberrant splicing in *STMN2* and *UNC13A* using ASOs.^42^

The first‐in‐human Phase 1 clinical trial, known as The ANQUR study (NCT05633459), has commenced to assess the safety, tolerability, and pharmacokinetics of QRL‐20 in patients with ALS without SOD1 and FUS mutations, aiming to rescue STMN2 expression.

It is noteworthy that nusinersen, an ASO drug designed to correct *SMN2* splicing defects, is utilized for the treatment motor neuron disease and spinal muscular atrophy.^43^ Therapeutic strategies employing splice‐switching oligonucleotides to restore defective genes are regarded as more physiologically rational treatments compared to knocking down target genes.

## CHCHD10

CHCHD10, a mitochondrial protein whose function remains unknown, has been proposed as a new candidate gene for ALS.^44^ CHCHD10‐related disease, which is extremely rare, presents with a variety of clinical findings, including mitochondrial myopathy, ALS, FTD, and spinal motor neuronopathy. Homozygous CHCHD10 knockout mice exhibit no remarkable phenotypes or mitochondrial abnormalities in the brain or skeletal muscle, suggesting that the mutant CHCHD10 may cause a toxic gain‐of‐function.^45^ A clinical trial of a personalized ASO drug designed for an individual participant is currently being conducted by the N‐Lorem Foundation, which aims to provide experimental treatments for very rare genetic disorders. (NCT06392126) This study is an N‐of‐1 trial, which uses a repeated crossover design in a single patient to allow direct comparison of treatment effects in rare diseases.

## Discussion

Initial attempts at vaccine therapy against amyloid‐β in Alzheimer's disease, much to the disappointment of neurologists, failed to prevent progressive cognitive impairment, despite achieving remarkable reductions in amyloid plaques.^46^ Since then, relentless research efforts have significantly advanced the field of Alzheimer's disease immunotherapy, and lecanemab has been fully approved by the FDA in the US and in Japan, becoming the first standard disease‐modifying drug for CNS proteinopathy. However, it is premature to draw conclusions regarding its benefits. Its efficacy appears limited to early Alzheimer's disease and does not improve cognitive function or stop the progression of neurodegeneration. Similarly, in ALS, ASO treatment has achieved proof‐of‐mechanism, but proof‐of‐concept in terms of clinical benefits is not yet definitive. Although ASO approaches are both reasonable and scientifically sound, a discernible clinical benefit has not always been observed in humans.

One explanation for why ASO‐mediated transcript knockdown cannot achieve proof‐of‐concept is that motor neuron loss in ALS is so advanced that, by disease onset, the motor system has already reached a state of no reserve capacity. As compensatory mechanisms do not work, improvement cannot be expected in the CNS, a non‐regenerative organ. This is supported by the knowledge that motor signs first appear in Parkinson's disease when the numbers of dopaminergic neurons in the substantia nigra are reduced to less than 50%.^47^

Another possibility is that a cascade of irreversible cell death due to toxic protein accumulation is already underway in the motor neurons at the early disease stage. Studies using patient stem cells and animal models of ALS have revealed disruption of the nuclear membrane/nuclear pore and associated DNA damage at early stages,^48^, ^49^ both of which represent formidable challenges for repair.^50^

Lastly, it is imperative to acknowledge that, thus far, phenotypes exhibited in knockout mice models that target genes associated with neurodegenerations have, at alarmingly high frequencies, appeared as adverse effects during human clinical trials. For example, skin cancer phenotypes were induced in both Presenilin knockout mice and when a γ secretase inhibitor was used,^51^, ^52^ reduced spine density and altered synaptic plasticity were observed in Bace1 knockout mice,^53^ and cognitive worsening and brain atrophy occurred after treatment with BACE1 inhibitors.^54^, ^55^ HTT, as described above, has possibly similar manifestations. This is because motor axonopathy and memory deficits have been observed in SOD1 and C9orf72 knockout mice, respectively, in an age‐dependent manner. Consequently, prudent vigilance must be exercised during long‐term treatment of ASO‐mediated knockdowns.

It is important to note that the transcription levels of causative genes in patients with ALS are not increased, with the exception of variants at the 3′ untranslated region of *FUS*, which contribute to the increase of *FUS* mRNA transcription.^56^, ^57^ It is conceivable that the reservoir of physiologically intact protein levels undergoes depletion, ensnared by the aggregation of misfolded protein. Although no phenotype is discernible in heterozygous targeted mice and humans, the reduced mRNA expression after ASO application in patients with proteinopathies can potentially engender on‐target adverse effects stemming from loss of function. Allele‐selective approaches using ASOs for silencing or genome editing against target mutations are therefore necessary to minimize reductions in intact protein expression. Allele‐selective ASOs have been used to target gain‐of‐toxic function mutations and single‐nucleotide polymorphisms (SNPs) in conjunction with aberrant transcripts.^58^, ^59^ For example, the PRECISION‐HD trials by Wave Life Sciences aim to selectively reduce the production of the mutant HTT by allele‐selective targeting of specific SNPs associated with the disease‐causing expanded CAG.^9^

There may be numerous references available for designing clinical trials based on preceding antibody therapies targeting the same proteinopathies. Most recent global clinical trials evaluating the efficacy of disease‐modifying drugs in Alzheimer's disease have shifted their target from dementia to preclinical Alzheimer's disease (ClinicalTrials.gov Identifier: NCT04468659 and NCT05026866).^60^ This shift underscores the rationale that ALS should similarly be diagnosed before symptom onset. This will require the identification of sensitive biomarkers or candidate risk genes to facilitate detection of preclinical ALS, enabling early therapeutic intervention before the irreversible cascade of neuronal death. A recent paper^61^, ^62^ described elevated levels of plasma extracellular vesicle TDP‐43, which is higher in ALS and FTD, and cryptic HDGFL2, a loss‐dependent cryptic epitope, as promising diagnostic biomarkers for ALS. These fluid biomarkers will allow noninvasive, faster and more accurate diagnosis of early ALS, as well as determining staging of the disease and the efficacy of drug treatments. These findings suggest that pre‐emptive treatment may hold promise.

## Author Contributions

Conception by DI. Manuscript preparation and editing by DI and KO.

## Funding Information

DI is supported by the Ministry of Education, Culture, Sports, Science and Technology of Japan (No. 21H02812).

## Conflict of Interest

The authors report no competing interests.

## Patient Consent for Publication

Not applicable.

## Data Availability

Not applicable.
